# Spontaneous resting‐state gamma oscillations are not predictive of autistic traits in the general population

**DOI:** 10.1111/ejn.13973

**Published:** 2018-06-19

**Authors:** Kristel De Groot, Jan W. Van Strien

**Affiliations:** ^1^ Erasmus School of Social and Behavioural Sciences Institute of Psychology Erasmus University Rotterdam Rotterdam The Netherlands; ^2^ Department of Applied Economics Erasmus School of Economics Erasmus University Rotterdam Rotterdam The Netherlands; ^3^ Erasmus University Rotterdam Institute for Behaviour and Biology (EURIBEB) Erasmus University Rotterdam Rotterdam The Netherlands

**Keywords:** autism spectrum disorder, autism‐spectrum quotient, broad autism phenotype, gamma activity, social functioning

## Abstract

The autism spectrum hypothesis states that not only diagnosed individuals but also individuals from the general population exhibit a certain amount of autistic traits. While this idea is supported by neuroimaging studies, there have been few electrophysiological studies. In particular, there have been no spontaneous resting‐state studies yet. In order to examine the autism spectrum hypothesis, the present study tried to predict the level of autistic traits typically developing young adults (*n* = 93) exhibit from spontaneous resting‐state gamma power, a measure that has been linked to social functioning impairments seen in autism. The influence of age and gender was controlled for by employing regression. It was expected that enhanced gamma activity would be predictive of self‐reported autistic traits. The model with only age and gender included reached significance, with higher age within this student population being related to more autistic traits. However, no relationship between either low (30–50 Hz) or high (50–70 Hz) gamma power and autistic traits was found. Models with eyes closed low gamma asymmetry and eyes closed high gamma asymmetry included did reach significance, but these findings were not robust, and the gamma asymmetry explained very little additional variance above age and gender. In addition, exploratory correlation analyses showed no relationship between the other power spectra (delta, theta, alpha and beta) on the one hand and autistic traits on the other hand, suggesting that any relationship between spontaneous resting‐state brain electrophysiology and autistic traits might not be strong enough to be detected in the general population.

## Introduction

Autism spectrum disorder (ASD) is a pervasive neurodevelopmental syndrome that is characterised by social communicational deficits, restricted and repetitive behaviour, preference for sameness and routines, and sensory abnormalities (American Psychiatric Association, 2013). One of most marked impairments concerns the social domain, which encompasses all verbal and nonverbal skills individuals use to interact with others, such as verbal reciprocity, eye contact, active listening and body language. The impairments in the social domain are considered to be of such importance for the disorder, that the triad of impairments is sometimes described as social communication, social interaction and social imagination, thereby implying that functioning in these subdomains is not necessarily completely disturbed, but that the impairment is specific to those actions that include social stimuli (Ten Eycke & Müller, 2015).

While ASD is diagnosed in a categorical manner, the idea that the disorder exists on a continuum is receiving more and more attention (Baron‐Cohen, Wheelwright, Skinner, Martin, & Clubley, 2001). This so‐called autism spectrum hypothesis states that not only diagnosed individuals but also individuals from the general population exhibit a certain amount of autistic traits. Individuals who show many traits might receive a diagnostic label. However, these diagnosed people do not qualitatively but only quantitatively differ from nondiagnosed individuals (Bailey, Palferman, Heavey, & Le Couteur, 1998; Baron‐Cohen et al., 2001; Hoekstra, Bartels, Cath, & Boomsma, 2008; Wing, 1988).

An important tool for examining the autism spectrum hypothesis is the Autism‐Spectrum Quotient (AQ), a brief, self‐administered instrument for measuring the degree to which an adult of normal intelligence possesses autistic traits (Baron‐Cohen et al., 2001). While the questionnaire has been used to relate autistic traits in typically developing (TD) individuals to behavioural outcomes, AQ scores have not been related much to brain outcomes. Some recent efforts for linking autistic traits in TD individuals to brain outcomes involved in social functioning were carried out with the use of neuroimaging techniques. The most important structures related to social functioning are the prefrontal cortex (PFC) and the superior temporal sulcus (STS). Studies show that less activity and lower density in these regions are related to social deficits in the clinical ASD population (Sugranyes, Kyriakopoulos, Corrigall, Taylor, & Frangou, 2011; Zilbovicius et al., 2006). In support of the spectrum view, studies showed that these brain characteristics are to a lesser extent also present in the general population, correlating with AQ scores across TD individuals. In particular, a near‐infrared spectroscopy (NIRS) study showed that AQ scores were negatively correlated with cerebral blood volume increase in the STS during face‐to‐face conversation (Suda et al., 2011). This is in line with a functional magnetic resonance imaging (fMRI) study showing that higher AQ scores were associated with decreased white matter volume and deactivation of the STS (Von dem Hagen et al., 2011). In a more direct comparison of a clinical and nonclinical group, Hosokawa, Nakadoi, Watanabe, Sumitani, and Ohmori (2015) applied a research paradigm to TD individuals that had previously been used on individuals with ASD. Their results showed that the degree of autistic traits people exhibited negatively correlated with the hemodynamic change the PFC showed in response to negative facial expressions. This weaker response was related to more difficulty in recognising these expressions. The impairment was milder but qualitatively similar to the findings from the clinical group (Nakadoi et al., 2012), thereby supporting the existence of a broad autism spectrum.

In contrast to the discussed neuroimaging studies, electrophysiological methods such as electroencephalography (EEG) have been employed only sparsely to examine the possibility of a broad autism spectrum. In particular, some EEG measures, such as spontaneous resting‐state activity, have not been employed at all in this area of research. Based on the suggestion that ASD is a connectivity disorder (Strzelecka, 2014), it is reasonable to assume that this cortical activation measure shows distinctive patterns for symptoms found in individuals with ASD and, extending this, in TD individuals with qualitatively similar but milder symptoms. As problems in the social domain are the most marked impairments found in ASD, it seems logical to examine electrophysiological patterns that are indicative of social functioning.

In EEG research on identifying an activation pattern related to social functioning, especially spontaneous gamma band activity has received attention. Gamma consists of waves within the 30–70 Hz frequency. Besides being linked to higher cognitive functions such as perceptual binding, gamma waves play an important role in the synchronisation of cortical networks. Although spontaneous gamma activity in ASD has been studied less than activity in the lower frequency bands (Rojas & Wilson, 2014), there is a vast set of recent studies showing that individuals with ASD have increased spontaneous gamma oscillations (Cornew, Roberts, Blaskey, & Edgar, 2012; Lushchekina, Podreznaya, Lushchekin, Novototskii‐Vlasov, & Strelets, 2013; Lushchekina, Podreznaya, Lushchekin, & Strelets, 2012; Orekhova et al., 2007; Van Diessen, Senders, Jansen, Boersma, & Bruining, 2015). Enhanced gamma band activity can therefore be considered a biomarker. In addition, other forms of gamma (reduced stimulus‐related oscillations) have already been observed in unaffected relatives of individuals with ASD (Rojas & Wilson, 2014; Rojas et al., 2011). This has two implications. First, it suggests that gamma band dysfunction is not only a biomarker but an endophenotype as well. Second, it means that gamma oscillations are suitable to be examined in light of a broad autism spectrum.

The underlying neurobiological mechanisms of cortical gamma function are well‐studied. A widely accepted mechanistic model for disrupted gamma generation concerns E/I imbalance: a disparity between excitatory (E) and inhibitory (I) activity (Gonzalez‐Burgos & Lewis, 2008; Port et al., 2015; Uhlhaas & Singer, 2010). Gamma oscillations emerge from coordinated interaction of excitation and inhibition onto pyramidal cells, which causes respectively increases and decreases in neuronal firing and synchronisation of cortical circuits (Buzsáki & Wang, 2012; Port et al., 2015; Rojas & Wilson, 2014; Uhlhaas & Singer, 2010). This mechanistic model forms the centre of several theoretical accounts of autism aetiology that state that suppressed GABAergic inhibition is a common factor in ASD and that high levels of excitatory (glutamatergic) compared to inhibitory (GABAergic) activity might even be involved in the pathogenesis of the disorder (Hussman, 2001; Rubenstein & Merzenich, 2003). The mechanistic model and its theoretical background are extended and supported by studies in which individuals with ASD show deviant GABA and glutamate levels and a disrupted balance between these two (Harada et al., 2011; Hussman, 2001). In addition, ASD often co‐occurs with seizure disorders, which are linked to an E/I imbalance as well (Brooks‐Kayal, 2010). As such, enhanced gamma activity can be explained by a theoretically defined biological mechanism of which central components have been proven to be altered in ASD.

Strong support for the idea of enhanced gamma band power as a broad endophenotype for the social impairments seen in ASD is offered by linking E/I imbalance, increased gamma power and social impairments. This is often done in rodent studies using the three‐chamber test, in which a mouse is placed in the middle of a three‐chambered box with openings between the chambers, and an empty cup in each of the outer chambers. Sociability is then tested by placing a conspecific mouse under one of the cups; social novelty preference is examined by placing a new mouse under the other cup. One study using the test examined knock‐out mice with an E/I imbalance as a result of mitochondrial dysfunction in parvalbuminergic interneurons, which are vital for maintaining the cortical E/I balance (Inan et al., 2016). As would be expected based on the discussed mechanisms, knock‐out mice exhibited increased gamma power and accompanying behavioural alterations: compared to control mice, they spent less time with a stranger mouse and did not prefer the conspecific to the empty cup.

The validity of enhanced gamma oscillatory activity as an endophenotype is further supported by studies showing that increased spontaneous gamma power and the accompanying social deficits are ameliorated by pharmacological modulation of the E/I balance. As gamma oscillations and impaired social information processing in ASD are most strongly linked to the firing of inhibitory GABAergic interneurons, development of pharmacological agents that restore the E/I imbalance has focused mostly on drugs that increase neuronal inhibition (Frye, 2014; Gonzalez‐Burgos & Lewis, 2008; Uhlhaas & Singer, 2010). One way of enhancing neuronal inhibition is with the use of benzodiazepines, which increase inhibitory neurotransmission through positive allosteric modulation of postsynaptic GABA‐A receptors. The impact of this on social behaviour was confirmed in a mouse model of autism in which clonazepam increased inhibitory neurotransmission in knock‐out mice, which ameliorated social deficits indicated by increased time spent with conspecifics as compared to an empty cup (Han, Tai, Jones, Scheuer, & Catterall, 2014). Another drug that has received much research attention is the muscle relaxant baclofen and its derivative STX209, the active R‐enantiomer arbaclofen (Frye, 2014; Hopkins, 2011). Postsynaptically, arbaclofen has a selective agonistic impact on GABA‐B receptors, thereby supporting inhibitory firing. Presynaptically, the agent inhibits the release of glutamate into the synaptic cleft, thereby blocking downstream signalling of glutamate receptors. The positive effect of arbaclofen on social functioning has been demonstrated in rodent studies using a mouse model of the FMR1 gene mutation that causes Fragile X Syndrome (FXS). Using this model, Silverman et al. (2015) showed that arbaclofen corrected synaptic abnormalities seen in FXS and ASD alike and that it reversed social approach deficits. When treated with only saline, knock‐out mice spent approximately equal time with the empty cup and a novel mouse. Administration of arbaclofen reversed this sociability deficit.

These findings are in accordance with mouse models in which *N*‐methyl‐d‐aspartate receptor (NMDAR) function is altered, causing an increase in circuit excitability. This disruption of the E/I balance was linked to gamma band abnormalities and created ASD‐like social novelty preference impairments that were reversed by administration of baclofen (Gandal et al., 2012). Similar findings were reported in research on schizophrenia, although modulation of GABAergic signalling with the use of baclofen only normalised gamma power but did not have corresponding effects on sociability (Billingslea et al., 2014). In contrast to this latter finding, a study using optogenetic activation of inhibitory cell excitability did show amelioration of social deficits (Yizhar et al., 2011). Evidence from clinical trials on humans is more limited at the present time, but follows the same line as the discussed rodent studies. Individuals with ASD show higher glutamine and lower GABA levels, both of which are associated with greater impairments in social cognition (Cochran et al., 2015). In addition, phase II trials show that administration of arbaclofen to individuals with ASD and FXS changes the E/I balance and improves social functioning (Berry‐Kravis et al., 2012; Erickson et al., 2014).

Approaching the subject from the other side, knowledge of the link between E/I imbalance, enhanced spontaneous gamma band activity, and social functioning can also contribute to evaluating the effect of both pharmacological and behavioural interventions. A recent study confirmed this by showing how a social skill intervention changed atypical gamma activity in individuals with ASD, thereby ameliorating clinical impairments (Vaughan Van Hecke et al., 2015). The study recorded spontaneous spectral power in a group TD adolescents and a group adolescents diagnosed with ASD. Spectral power was recorded again after half of the ASD group had received a 14‐week social skills training, the Program for the Education and Enrichment of Relational Skills (PEERS). Prior to the intervention, both groups of participants with ASD exhibited a specific pattern of lower left‐dominant asymmetry in the beta and lower gamma band compared to TD individuals. Following the intervention, the experimental ASD group significantly increased in left‐dominant asymmetry, which was specific to the gamma band. The resulting gamma band pattern had changed to resemble the TD gamma band pattern. The waiting list group that did not receive PEERS did not show any changes. Most important, the normalised gamma band pattern was related to more social skill knowledge, better social contacts and less social problems, hereby supporting the relationship between spontaneous gamma oscillations and social functioning. These findings do not attain to generally increased power, but show hemispheric asymmetry, thereby suggesting that rebalancing atypical gamma may be less straightforward than only establishing lower power values. It does, however, confirm the link between spontaneous gamma power and social symptoms found in ASD.

The discussed rodent studies, clinical trials and intervention data show how rebalancing high spontaneous (right hemisphere) gamma oscillations is accompanied by amelioration of social deficits. However, this link has not yet been examined in the general population. Doing so does make sense though because impaired social functioning is a defining characteristic of autism. If the autism spectrum hypothesis is true for oscillatory data, TD individuals with higher spontaneous resting‐state gamma band power should exhibit more autistic traits as measured by the AQ. The present study examines this, hereby aiming at extending the findings on the relationship between enhanced gamma power and autistic traits into the general population. For the sake of completeness, and to take the findings from Vaughan Van Hecke et al. (2015) into account, asymmetry scores will also be examined, hypothesising lower left‐dominant gamma asymmetry as being predictive of more autistic traits.

Because spontaneous resting‐state oscillatory data in general have not been related to autistic traits in the TD population, power spectra other than gamma will be analysed in an exploratory way. The predicted relationship between these spectra and autistic traits is related to the discussed E/I imbalance found in ASD, which has shown to lead to a U‐shaped oscillatory pattern in which ASD is characterised by enhanced low‐frequency (delta, theta) and high‐frequency (beta, gamma) activity and reduced mid‐range (alpha) power (Wang et al., 2013). We hypothesise that these ASD patterns will be found in high‐scoring TD individuals as well, thereby supporting the spectrum view of autism.

## Materials and methods

### Participants

Participants were 93 TD students from the Institute of Psychology of the Erasmus University Rotterdam who participated in exchange for course credit. A power analysis determined that this sample size was appropriate to achieve a medium‐sized two‐tailed power (1 − β) > 0.85 for the main analyses (*α* = 0.05). The sample consisted of 36 male and 57 female participants with a mean age of *M* = 21.03 (*SD* = 2.37), range 18–30 years. Participants were informed about the nature of the measurements (EEG) beforehand, and written informed consent was obtained from all participants included in the study. All procedures performed were in accordance with the ethics standards of the institutional review board and with the 1964 Helsinki declaration and its later amendments.

### Autism‐spectrum quotient

The Autism‐Spectrum Quotient (AQ, Baron‐Cohen et al., 2001) is a continuous, quantitative self‐report measure of autistic traits in adults of normal intelligence. The questionnaire consists of 50 questions, divided into 5 subscales of 10 items each: social skill, attention switching, attention to detail, communication and imagination. Items are answered on a 4‐point Likert scale: definitely agree, slightly agree, slightly disagree, definitely disagree. Completing all items takes approximately 10 min. Both the original English version of the test and its Dutch translation show satisfactory psychometric properties (Baron‐Cohen et al., 2001; Hoekstra et al., 2008). The original scoring scheme as proposed by Baron‐Cohen et al. (2001) is binary, ignoring the degree of agreement or disagreement. In line with Austin (2005) and Hoekstra et al. (2008), we included all four levels in scoring, which yields higher internal consistency and test‐retest reliabilities than binary scoring (Stevenson & Hart, 2017), and which has been shown to improve the reliable range of measurement significantly (Murray, Booth, McKenzie, & Kuenssberg, 2016). This resulted in a minimum total score of 50 (the individual reports having no autistic traits) and a maximum score of 200 (the individual reports having the full range of autistic traits). As could be expected based on score variability, reliability was better when using the full‐range scoring scheme. Cronbach's alpha was *α* = 0.85 for the composite score (as opposed to *α* = 0.78 using binary scores), *α* = 0.76 (*α* = 0.56) for social skill, *α* = 0.76 (*α* = 0.67) for attention switching, *α* = 0.62 (*α* = 0.53) for attention to detail, *α* = 0.67 (*α* = 0.59) for communication and *α* = 0.57 (*α* = 0.50) for imagination.

### Procedure

The measures were part of a larger study examining both behavioural and brain correlates of impairments often seen in ASD. The total experimental session took approximately 90 min, including breaks. Sessions either started at 9:00 am, 11:00 am, 1:00 pm, or 3:00 pm. After arrival, participants first filled out three questionnaires, including the AQ. Thereafter, the participant was seated in a comfortable chair in a light and sound‐attenuated EEG room. The electrodes were placed, and a brief instruction about the task was given. Then, spontaneous resting‐state EEGs were recorded. Previous studies recorded this while participants either focused on a fixation point of some sort (Orekhova et al., 2007; Vaughan Van Hecke et al., 2015) or while participants had their eyes closed (Cornew et al., 2012; Lushchekina et al., 2012, 2013; Van Diessen et al., 2015). To cover both, two continuous resting‐state EEGs were measured, each with a 180 s duration. This duration is approximately similar to that used in previous studies [e.g., two epochs of 120 s each by Orekhova et al. (2007) and one epoch of 180 s by Vaughan Van Hecke et al. (2015)]. Before each measurement period, a short text reminding the participant to be relaxed and to either have his or her eyes open or closed appeared on the screen. The order in which the conditions were presented was counterbalanced across participants.

### Electrophysiological recordings and signal processing

Electroencephalography was recorded using a 32‐channel amplifier and ActiveTwo data acquisition software (Biosemi, Amsterdam, the Netherlands). A number of 32 Ag/AgCl active electrodes were placed on the scalp by means of a head cap according to the 10–20 placing system. The electro‐oculogram (EOG) was recorded by placing flat electrodes above and below the left eye (vertical EOG) and at the outer canthi of both eyes (horizontal EOG). Referencing was carried out via two electrodes placed on the mastoids. All signals were digitised with a sampling rate of 512 Hz.

The data were analysed offline with BrainVision Analyzer 2 (Brain Products, Gilching, Germany). All EEG channels were referenced to the mathematically linked mastoid electrodes. A low cut‐off of 0.1 Hz was applied, together with a notch filter of 50 Hz to filter out artefact caused by electrical power lines. The epochs for each condition were divided into 179 one‐second segments. Ocular artefact correction was performed using the Gratton and Coles algorithm (Gratton, Coles, & Donchin, 1983), an offline Eye Movement Correction Procedure (EMCP) that first removes stimulus‐linked variability from the EOG and EEG traces. It then uses the EOG and EEG records to estimate two propagation factors (one for blinks and one for eye movements), which describe the relationship between both traces. Finally, it uses these propagation factors to correct for ocular artefacts. The advantage of employing the Gratton and Coles algorithm is threefold. First, it corrects for but does not discard trials containing ocular artefact, thereby retaining more trials. Second, blinks and eye movements are corrected for separately by means of the two factors. Third, the calculation of the propagation factors is based on data from the experimental session itself rather than on data from, for example, a calibration session.

Automatic artefact rejection allowed a maximal difference of 200 μV within each segment. Seven participants had channels on which more than 20% of the 179 segments per condition was removed due to artefacts. Their data were therefore excluded from all analyses, leaving the 93 participants whose characteristics were described before. Finally, absolute power (μV^2^) was calculated by applying a Fast Fourier Transformation (FFT) onto the full spectrum, using a periodic Hanning window of 10% with variance correction. During the FFT algorithm, signals are transformed from the time domain to the frequency domain, dividing them into frequency components that all have their own distinctive amplitude and phase. The FFTs were averaged to compute the band power of delta (0–4 Hz), theta (4–8 Hz), alpha (8–12 Hz), beta (12–30 Hz), low gamma (30–50 Hz) and high gamma (50–70 Hz). Although the closed eyes condition resulted in stronger alpha waves compared to the open eyes condition, the power spectra per frequency were relatively homogeneously distributed across the scalp.

Especially high‐frequency EEG (i.e., beta and gamma) is susceptible to myogenic artefacts. In previous research, the lowest beta and gamma spectral power has been observed at the midline, suggesting lower contamination of myogenic artefacts in those regions (Orekhova et al., 2007). Therefore, some studies advise focusing exclusively on the midline electrodes to minimise the contribution of myogenic artefacts. To include a broader range, we extended this to adjacent electrodes as well. Oscillatory activity was therefore pooled from the midline and adjacent electrodes: a frontal cluster (F3, Fz, F4), a central cluster (C3, Cz, C4), a parietal cluster (P3, Pz, P4) and an occipital cluster (O1, Oz, O2). As mentioned before, there were no significant topographical differences across the scalp for most frequency bands. Therefore, it was decided to analyse the clusters together.

### Analyses

After preprocessing the data, the final analyses were performed using IBM SPSS version 24. Five sets of analyses were run, all using a Bonferroni‐corrected alpha level of *α* = 0.05. Analyses involving spectral data were made robust against their inherent skewness by using bootstrapping (1,000 samples), a technique that estimates the sampling distribution of a statistic by taking repeated samples from a dataset (Efron, 1987). First, the descriptive statistics of both the main dependent (AQ score) and independent (low and high gamma power) variable were described, and it was examined whether both differed between gender (by means of an independent samples *t*‐test) and across age (by means of a correlation analysis). For the AQ, the binary scoring procedure from Baron‐Cohen et al. (2001) was used to examine whether any participants scored above the clinical cut‐off. Second, we examined whether gamma activity predicted self‐reported autistic traits. To this end, we ran four hierarchical multiple regression analyses (HMRAs): one predicting AQ score from the lower gamma band as measured with the eyes open, one predicting AQ score from the lower gamma band as measured with the eyes closed, one predicting AQ score from the higher gamma band as measured with the eyes open and one predicting AQ score from the higher gamma band as measured with the eyes closed. All regressions controlled for the influence of age and gender by hierarchically entering the variables into the model. Third, we examined whether gamma asymmetry values predicted self‐reported autistic traits. To this end, we ran the exact same regression analyses as described above, but then with the gamma asymmetry values as predictors. Asymmetry scores were calculated by subtracting the average value of the used right hemisphere electrodes (F4, C4, P4, O2) from the average value of the used left hemisphere electrodes (F3, C3, P3, O1). Fourth, an exploratory correlation analysis was executed in which all frequency bands (averaged across eyes open and closed) and all AQ (sub)scores were correlated with each other. Fifth, a robustness check was performed. EEG (pre)processing requires many analytical decisions. In the robustness check, it was examined whether the findings changed as a result of critical alternative decisions.

## Results

### Descriptive statistics

#### The autism‐spectrum quotient

The mean AQ score was *M* = 98.76 (*SD* = 13.71), range 65–134. The distribution was approximately normal as determined by visual inspection of the normal Q‐Q plot. This was supported by the results from the Kolmogorov–Smirnov test, *D*(93) = 0.08, *p* = 0.200. AQ score did not significantly differ between men (*M = *101.92) and women (*M* = 96.77), *t*(91) = 1.78, *p* = 0.078, η^2^_p_ = 0.03. AQ score did significantly positively correlate with age, *r* = 0.35, *p* = 0.001. None of the participants scored above the clinical cut‐off score of 32 (Baron‐Cohen et al., 2001) as determined by the binary scoring scheme (present range 2–29 of 0–50).

#### Gamma power

The mean power was *M* = 0.23 (*SD* = 0.13, range 0.08–0.85) for eyes open low gamma, *M* = 0.18 (*SD* = 0.13, range 0.05–0.95) for eyes closed low gamma, *M* = 0.14 (*SD* = 0.09, range 0.03–0.48) for eyes open high gamma and *M* = 0.11 (*SD* = 0.09, range 0.02–0.51) for eyes closed high gamma. All distributions were positively skewed as determined by visual inspection of the normal Q‐Q plots. These violations of normality were confirmed by the results from Kolmogorov–Smirnov tests, *D*(93) = 0.14, *p *<* *0.001 (eyes open low gamma), *D*(93) = 0.16, *p *<* *0.001 (eyes closed low gamma), *D*(93) = 0.17, *p *<* *0.001 (eyes open high gamma) and *D*(93) = 0.17, *p *<* *0.001 (eyes closed high gamma). None of the mean values significantly differed between men and women, *t*(91) = 0.19, *p* = 0.844, η^2^_p_ < 0.01 (eyes open low gamma), *t*(91) = −0.03, *p* = 0.974, η^2^_p_ < 0.01 (eyes closed low gamma), *t*(91) = 0.63, *p* = 0.508, η^2^_p_ < 0.01 (eyes open high gamma) and *t*(91) = 0.37, *p* = 0.699, η^2^_p_ < 0.01 (eyes closed high gamma). Finally, power did not significantly correlate with age, *r* = 0.07 (bootstrap bias 0.011), *p* = 0.521 (eyes open low gamma), *r* = 0.11 (bootstrap bias 0.017), *p* = 0.318 (eyes closed low gamma), *r* = 0.10 (bootstrap bias 0.008), *p* = 0.353 (eyes open high gamma) and *r* = 0.11 (bootstrap bias 0.009), *p* = 0.284 (eyes closed high gamma).

### Gamma power regression analyses

#### Does eyes open low gamma power predict the amount of autistic traits in TD individuals, controlling for age and gender?

The HMRA showed that age and gender together significantly contributed to the regression model, *F*_2,90_ = 6.85, *p* = 0.002, *R* ^2^ = 0.13. A model with eyes open low gamma power included as well also reached significance, *F*_3,89_ = 5.26, *p* = 0.002, *R* ^2^ = 0.15. The inclusion of gamma power in the model explained an additional 1.84% of variance, which was not significant, *F*_change_(1, 89) = 1.93, *p* = 0.168. In both models, only one predictor significantly contributed to the prediction: age (*p* = 0.004 in both models), with older individuals exhibiting more autistic traits. The predictor coefficients of both models and the bias‐corrected and accelerated (BCa) confidence intervals (CIs) are shown in Table 1.

**Table 1:** Bootstrapped hierarchical multiple regression models predicting AQ score from gamma power

		*b*	*p*	95% BCa CI
Eyes open low gamma	Age	1.89	0.004	0.65 to 3.40
	Gender	−2.68	0.319	−7.62 to 2.59
	Age	1.89	0.009	0.67 to 3.39
	Gender	−2.69	0.329	−7.92 to 2.60
	Gamma	−14.08	0.098	−31.20 to 7.42
Eyes closed low gamma	Age	1.89	0.009	0.67 to 3.43
	Gender	−2.68	0.344	−8.24 to 2.73
	Age	1.96	0.008	0.72 to 3.42
	Gender	−2.57	0.366	−8.04 to 2.95
	Gamma	−11.73	0.107	−28.60 to 3.19
Eyes open high gamma	Age	1.89	0.007	0.68 to 3.43
	Gender	−2.68	0.312	−8.46 to 3.06
	Age	1.94	0.008	0.69 to 3.55
	Gender	−2.81	0.295	−8.77 to 3.01
	Gamma	−16.36	0.235	−42.85 to 16.86
Eyes closed high gamma	Age	1.89	0.008	0.65 to 3.30
	Gender	−2.68	0.309	−8.43 to 2.52
	Age	1.98	0.003	0.70 to 3.40
	Gender	−2.72	0.331	−8.37 to 2.53
	Gamma	−21.97	0.071	−49.85 to 1.09

Notes

*N* = 93.

BCa CI: bias‐corrected and accelerated confidence interval.

#### Does eyes closed low gamma power predict the amount of autistic traits in TD individuals, controlling for age and gender?

The HMRA showed that age and gender together significantly contributed to the regression model, *F*_2,90_ = 6.85, *p* = 0.002, *R* ^2^ = 0.13. A model with eyes closed low gamma power included as well also reached significance, *F*_3, 89_ = 5.03, *p* = 0.003, *R* ^2^ = 0.15. The inclusion of gamma power in the model explained an additional 1.27% of variance, which was not significant, *F*_change_(1, 89) = 1.32, *p* = 0.254. In both models, only one predictor significantly contributed to the prediction: age (*p* = 0.009 for model 1, *p* = 0.008 for model 2), with older individuals exhibiting more autistic traits. The predictor coefficients of both models and the BCa CIs are shown in Table 1.

#### Does eyes open high gamma power predict the amount of autistic traits in TD individuals, controlling for age and gender?

The HMRA showed that age and gender together significantly contributed to the regression model, *F*_2,90_ = 6.85, *p* = 0.002, *R* ^2^ = 0.13. A model with eyes open high gamma power included as well also reached significance, *F*_3,89_ = 4.99, *p* = 0.003, *R* ^2^ = 0.14. The inclusion of gamma power in the model explained an additional 1.19% of variance, which was not significant, *F*_change_(1, 89) = 1.24, *p* = 0.269. In both models, only one predictor significantly contributed to the prediction: age (*p* = 0.007 for model 1, *p* = 0.008 for model 2), with older individuals exhibiting more autistic traits. The predictor coefficients of both models and the BCa CIs are shown in Table 1.

#### Does eyes closed high gamma power predict the amount of autistic traits in TD individuals, controlling for age and gender?

The HMRA showed that age and gender together significantly contributed to the regression model, *F*_2,90_ = 6.85, *p* = 0.002, *R* ^2^ = 0.13. A model with eyes closed high gamma power included as well also reached significance, *F*_3,89_ = 5.38, *p* = 0.002, *R* ^2^ = 0.15. The inclusion of gamma power in the model explained an additional 2.13% of variance, which was not significant, *F*_change_(1, 89) = 2.24, *p* = 0.138. In both models, only one predictor significantly contributed to the prediction: age (*p* = 0.008 for model 1, *p* = 0.003 for model 2), with older individuals exhibiting more autistic traits. The predictor coefficients of both models and the BCa CIs are shown in Table 1.

### Gamma asymmetry regression analyses

#### Does eyes open low gamma asymmetry predict the amount of autistic traits in TD individuals, controlling for age and gender?

The HMRA showed that age and gender together significantly contributed to the regression model, *F*_2,90_ = 6.85, *p* = 0.002, *R* ^2^ = 0.13. A model with eyes open low gamma asymmetry included as well also reached significance, *F*_3,89_ = 4.83, *p* = 0.004, *R* ^2^ = 0.14. The inclusion of gamma asymmetry in the model explained an additional 0.79% of variance, which was not significant, *F*_change_(1, 89) = 0.81, *p* = 0.370. In both models, only one predictor significantly contributed to the prediction: age (*p* = 0.010 for model 1, *p* = 0.008 for model 2), with older individuals exhibiting more autistic traits. The predictor coefficients of both models and the BCa CIs are shown in Table 2.

**Table 2:** Bootstrapped hierarchical multiple regression models predicting AQ score from gamma asymmetry

		*b*	*p*	95% BCa CI
Eyes open low gamma asymmetry	Age	1.89	0.010	31.86 to 84.61
	Gender	−2.68	0.326	−8.50 to 2.70
	Age	1.95	0.008	0.73 to 3.49
	Gender	−2.35	0.395	−8.34 to 3.21
	Gamma	27.74	0.410	−44.22 to 88.04
Eyes closed low gamma asymmetry	Age	1.89	0.003	32.84 to 86.18
	Gender	−2.68	0.347	−7.75 to 2.61
	Age	2.07	0.007	0.95 to 3.51
	Gender	−2.20	0.421	−7.49 to 3.36
	Gamma	63.91	0.027	4.41 to 132.47
Eyes open high gamma asymmetry	Age	1.89	0.005	0.72 to 3.38
	Gender	−2.68	0.354	−8.13 to 2.66
	Age	1.92	0.004	0.70 to 3.43
	Gender	−2.59	0.380	−8.04 to 2.99
	Gamma	17.80	0.695	−79.53 to 117.03
Eyes closed high gamma asymmetry	Age	1.89	0.002	33.76 to 85.79
	Gender	−2.68	0.341	−7.58 to 1.82
	Age	2.05	0.008	0.72 to 3.66
	Gender	−2.35	0.399	−7.28 to 2.45
	Gamma	92.53	0.028	−3.62 to 184.410

Notes

*N* = 93.

BCa CI: bias‐corrected and accelerated confidence interval.

#### Does eyes closed low gamma asymmetry predict the amount of autistic traits in TD individuals, controlling for age and gender?

The HMRA showed that age and gender together significantly contributed to the regression model, *F*_2,90_ = 6.85, *p* = 0.002, *R* ^2^ = 0.13. A model with eyes closed low gamma asymmetry included as well also reached significance, *F*_3,89_ = 5.68, *p* = 0.001, *R* ^2^ = 0.16. The inclusion of gamma asymmetry in the model explained an additional 2.85% of variance, which was not significant, *F*_change_(1, 89) = 3.02, *p* = 0.086. In both models, age significantly contributed to the prediction (*p* = 0.003 for model 1, *p* = 0.007 for model 2), with older individuals exhibiting more autistic traits. In addition, eyes closed low gamma asymmetry significantly contributed to the prediction (*p* = 0.027), with higher asymmetry scores (higher left dominance) being related to more autistic traits. The predictor coefficients of both models and the BCa CIs are shown in Table 2.

#### Does eyes open high gamma asymmetry predict the amount of autistic traits in TD individuals, controlling for age and gender?

The HMRA showed that age and gender together significantly contributed to the regression model, *F*_2,90_ = 6.85, *p* = 0.002, *R* ^2^ = 0.13. A model with eyes open high gamma asymmetry included as well also reached significance, *F*_3,89_ = 4.60, *p* = 0.005, *R* ^2^ = 0.13. The inclusion of gamma asymmetry in the model explained an additional 0.21% of variance, which was not significant, *F*_change_(1, 89) = 0.21, *p* = 0.646. In both models, only one predictor significantly contributed to the prediction: age (*p* = 0.005 for model 1, *p* = 0.004 for model 2), with older individuals exhibiting more autistic traits. The predictor coefficients of both models and the BCa CIs are shown in Table 2.

#### Does eyes closed high gamma asymmetry predict the amount of autistic traits in TD individuals, controlling for age and gender?

The HMRA showed that age and gender together significantly contributed to the regression model, *F*_2,90_ = 6.85, *p* = 0.002, *R* ^2^ = 0.13. A model with eyes closed high gamma asymmetry included as well also reached significance, *F*_3,89_ = 6.85, *p* = 0.002, *R* ^2^ = 0.16. The inclusion of gamma asymmetry in the model explained an additional 2.96% of variance, which was not significant, *F*_change_(1, 89) = 3.14, *p* = 0.080. In both models, age significantly contributed to the prediction (*p* = 0.008 in both models), with older individuals exhibiting more autistic traits. In addition, eyes closed high gamma asymmetry significantly contributed to the prediction (*p* = 0.028), with higher asymmetry scores (higher left dominance) being related to more autistic traits. However, the BCa CI did include zero. The predictor coefficients of both models and the BCa CIs are shown in Table 2.

### Exploratory correlation analyses

A Pearson's correlation analysis was run including all frequency bands (averaged across eyes open and closed) and all AQ subscores. The correlation coefficients are shown in Table 3. Most AQ subscores correlated significantly and positively with each other. The least correlated AQ subscore was attention to detail. Several of the frequency bands correlated significantly and negatively with each other, except for the low and high gamma bands, which showed a very strong positive correlation. The AQ subscores did not significantly correlate with the frequency bands, with one exception, namely a significant positive correlation between the AQ social skill subscore and alpha power.

**Table 3:** Pearson's correlation matrix containing all frequency bands (averaged across eyes open and closed) and all AQ subscores

		1	2	3	4	5	6	7	8	9	10	11	12
1	AQ total	1											
2	AQ social	0.78**	1										
3	AQ change	0.78**	0.55**	1									
4	AQ communication	0.74**	0.59**	0.52**	1								
5	AQ imagination	0.71**	0.38**	0.41**	0.51**	1							
6	AQ detail	0.44**	0.16	0.16	−0.03	0.20*	1						
7	Delta	−0.03	−0.16	−0.10	0.02	0.02	0.15	1					
8	Theta	0.02	−0.06	0.05	0.09	0.08	−0.10	0.05	1				
9	Alpha	0.19	0.25*	0.12	0.19	0.10	0.02	−0.27*	0.05	1			
10	Beta	0.05	0.04	0.10	−0.03	0.05	0.01	−0.23*	−0.32**	−0.17	1		
11	Low gamma	−0.10	−0.11	−0.03	−0.18	−0.12	0.07	−0.15	−0.51**	−0.51**	0.14	1	
12	High gamma	−0.09	−0.08	−0.03	−0.13	−0.12	0.04	−0.17	−0.52**	−0.47**	0.06	0.97**	1

Note

*N* = 93.

*p < 0.05, **p < 0.01. For italicised correlations, the 95% bias‐corrected and accelerated (BCa) confidence interval (CI) did not contain zero.

### Robustness checks

Two robustness checks were run to examine whether the reported findings changed as a result of critical alternative analytical decisions in EEG preprocessing. First, it was examined whether the used data filters impacted the findings: the low cut‐off of 0.1 Hz and the notch filter of 50 Hz. With regard to the former, low‐frequency activity (such as 0.1 Hz) is theoretically possible, but unusual in individuals in a normal awake state. Therefore, the analyses were rerun using a higher low cut‐off: 0.5 Hz. None of conclusions changed. With regard to the latter, analyses were rerun without applying a notch filter. Although the filter removes artefact caused by electrical power lines, it inherently causes a distortion of oscillatory activity, particularly in the gamma band. However, reanalysing the data without the notch filter did not change the conclusions. Next to the filter robustness check, it was also examined whether the use of relative vs. absolute power had any impact on the results. Therefore, all analyses were rerun using relative instead of absolute power. Again, none of the main findings and hence none of the conclusions changed.

## Discussion

The present study examined whether spontaneous resting‐state gamma band power or asymmetry in TD individuals predicted the amount of autistic traits these individuals reported, controlling for the influence of age and gender. Based on neuroimaging studies, rodent studies, clinical trials and intervention data, we expected that higher spontaneous resting‐state gamma band power and possibly lower left‐dominant gamma asymmetry would be predictive of more autistic traits. However, the results showed that gamma power was not related to AQ score. In the models including gamma power, the only significant predictor of autistic symptoms was age, with higher age being related to more autistic traits. For gamma asymmetry, models including both eyes closed low (30–50 Hz) and high (50–70 Hz) gamma were predictive of autistic traits, with higher left‐dominant gamma asymmetry being predictive of more autistic traits. This was opposite to the hypotheses based on findings from Vaughan Van Hecke et al. (2015). In addition, the robustness of these findings is questionable. First, the BCa CI of the eyes closed high gamma asymmetry contained zero. Second, the additional variance explained by adding either low or high gamma asymmetry to the model was very small (<3% in both cases) and not significant. Therefore, the influence of gamma asymmetry in predicting autistic traits is questionable.

Because no previous research has related spontaneous resting‐state EEG data to autistic traits in TD individuals, the other power spectra were analysed as well in an exploratory manner. The hypotheses were based on studies showing a U‐shaped oscillatory pattern in which individuals with ASD have enhanced low‐frequency (delta, theta) and high‐frequency (beta, gamma) activity and reduced mid‐range (alpha) power (Wang et al., 2013). A correlation analysis showed that there were no significant relationships between AQ (sub)score(s) and either delta, theta, alpha, beta, low gamma, or high gamma. The only exception was a significant positive correlation between the AQ social skill subscore and alpha power. This was contrary to the expectation of reduced alpha power being related to more autistic traits.

To conclude, spontaneous resting‐state gamma power and asymmetry are no robust predictors of self‐reported autistic traits in a TD population. Therefore, enhanced spontaneous resting‐state gamma power cannot function as a broad endophenotype for the social impairments seen in ASD. These findings indicate that the relationship between spontaneous resting‐state gamma power and autistic traits is not present in TD individuals, and suggest that more extreme (clinical) scores are needed to establish this link. This is in line with a magnetic resonance imaging (MRI) study by Koolschijn, Geurts, Van Der Leij, and Scholte (2015) that did not find an association between autistic traits and brain morphometry in TD individuals, questioning the assumption of a broad endophenotype as well. The present lack of a relationship between spontaneous resting‐state oscillatory data and autism in a TD population is supported by the finding that not only gamma but almost none of the examined power spectra was found to be related to autistic traits in TD individuals, while they have all been related to ASD symptomatology in the clinical population (see Wang et al., 2013, for a review).

Taking all findings into account, one possible area future studies should focus on concerns the nonunitary nature of the gamma band construct (Rojas & Wilson, 2014). Although we found no link between autistic traits in a TD group and spontaneous resting‐state gamma, stimulus‐related gamma has been related to autistic traits in relatives of individuals with the disorder, thereby in fact supporting the autism spectrum hypothesis (Rojas & Wilson, 2014; Rojas et al., 2011). Therefore, it is possible that although the link between spontaneous resting‐state gamma power and autistic traits is not strong enough to appear in a TD group, stimulus‐related gamma activity is. Thus, future studies should examine if and how different gamma oscillations are related to autistic traits. A variation on this suggestion concerns examining brain connectivity, which differs across frequencies, with potential overconnectivity in the higher frequency bands of individuals diagnosed with ASD. Indeed, connectivity has been shown to be altered in clinical autism populations as measured with EEG/magnetoencephalography (MEG) (for an overview, see O'Reilly, Lewis, & Elsabbagh, 2017). So far, this is supported in a TD population using fMRI (Di Martino et al., 2009), leaving the relationship between electrophysiologically measured connectivity and autistic traits across the broad autism spectrum unknown. A second suggestion for future research concerns the demarcation of the gamma band. Most discussed studies operationalise gamma as frequencies between approximately 30 and 70 Hz (Lushchekina et al., 2012, 2013; Orekhova et al., 2007), although some use a lower upper limit of 45 Hz (Van Diessen et al., 2015) or 50 Hz (Vaughan Van Hecke et al., 2015). Higher frequencies are seldom examined (Cornew et al., 2012), and there is no consensus as to what the upper limit of gamma is (Rojas & Wilson, 2014). Higher frequencies are usually associated with pathological outcomes such as epilepsy, and as seizures have an increased prevalence in ASD (Brooks‐Kayal, 2010), higher frequencies might be increased in individuals scoring high on the autism spectrum (Rojas & Wilson, 2014).

In addition to these two possible follow‐up examinations, the present report also contains age‐related findings that are in need of follow‐up attention. First, higher age predicted reporting more autistic traits. While gender differences have often been examined for AQ scores, age has not received much attention. Hoekstra et al. (2008) examined the effect of both age and gender in a general population sample, showing no significant relationship between AQ scores and age. Similar findings were obtained by Ruzich et al. (2016). Other studies focusing on the (psychometric) characteristics of the AQ (e.g., Baron‐Cohen et al., 2001) do report mean group age, but do not separately analyse age differences in AQ. In addition, the mean group ages reported in those studies are very specific to the groups themselves, with general population samples and patient groups often having a higher mean age than students. This means that age and group are confounded: the groups are inherently different on clinical diagnosis and often as well on variables such as intelligence and comorbidity, making it difficult to draw conclusions about the effect of age on autistic traits. In addition, most studies on ASD focus on children, paying little attention to the development of diagnosed individuals in (young) adulthood and thereafter. The present findings suggest the existence of a relationship between age and autistic traits in young TD adults. This outcome could be a by‐product of methodological and statistical characteristics of the data. With regard to the former, the relationship between age and AQ could be explained by the sample itself. The higher level of autistic traits in older students could be a consequence of general differences in cognition and/or personality in this group. This is a common pitfall encountered when using cross‐sectional data. With regard to the latter, thorough examination of the data shows that the relationship between age and AQ is characterised by heteroscedasticity, which is not necessarily bad, but which could invalidate the fitted models as they assume equal outcome variance across different values of the predictor. Together with the limited previous research on this matter, these problems make further investigation into the relationship between age and AQ essential.

A second age‐related suggestion for future research concerns the EEG studies. Just as in the case of behavioural studies, most research on (spontaneous resting‐state) EEG in autism focuses on children. This is also true for many of the studies from which the hypotheses as postulated in the current study were derived (Cornew et al., 2012; Lushchekina et al., 2012, 2013; Orekhova et al., 2007; Van Diessen et al., 2015; Vaughan Van Hecke et al., 2015). Deriving hypotheses about adults from studies carried out on children requires the assumption of no major discrepancies between those two groups on the variable of interest. Because of the lack of studies on spontaneous resting‐state EEG in adults with ASD, it is difficult to determine whether the findings from children can be extrapolated to an older population. There is some evidence indicating that this cannot be done in a straightforward way. Barriga‐Paulino, Flores, and Gómez (2011) showed that children (aged 8–13) have higher spectral power in lower frequency bands (delta, theta), while young adults (aged 18–23) have higher spectral power in middle to higher frequency bands (alpha, beta). Examining a more restricted age range, Barry and Clarke (2009) showed similar findings: across age 8–12, delta and theta activity decreased, while alpha and beta activity increased with every 1‐year interval. This was true for both absolute and relative power. Although both studies did not examine gamma band activity and did only look at TD individuals, the found pattern suggests that EEG frequency hypotheses concerning adults obtained from studies on children should be approached with caution. This is especially true when findings are opposite to what was expected based on child studies, such as the positive relationship between spontaneous resting‐state alpha power and autistic traits found in the present study. In addition, it illustrates the need for studies on adults who score high on the autism spectrum. Although autism does not disappear at age 12, most research on it does.

## Ethical approval

*Written* informed consent was obtained from all participants included in the study. The research proposal was reviewed by the Ethical Committee of the Department of Psychology, Education & Child Studies of the Erasmus University Rotterdam. All procedures performed were in accordance with the 1964 Helsinki Declaration and its later amendments.

## Conflict of interest

Kristel de Groot and Jan van Strien declare that they have no conflict of interest.

## Data accessibility

All data are archived via Surfdrive and will be shared upon request.

## Author contributions

KDG conceived of the study, participated in its design, performed most measurements and statistical analyses and interpreted the data. JVS participated in the design and coordination of the study, participated in analysing the data and revised the manuscript critically for important intellectual content. All authors read and approved the final manuscript.

**Abbreviations**
AQ: Autism‐spectrum quotient
ASD: Autism spectrum disorder
BCa: Bias‐corrected and accelerated
CI: Confidence interval
EEG: Electroencephalography
EMCP: Eye movement correction procedure
fMRI: Functional magnetic resonance imaging
FXS: Fragile X syndrome
HMRA: Hierarchical multiple regression analysis
IBM: International business machines corporation
MEG: Magnetoencephalography
MRI: Magnetic resonance imaging
NIRS: Near‐infrared spectroscopy
NMDAR: *N*‐methyl‐d‐aspartate receptor
PEERS: Program for the Education and Enrichment of Relational Skills
PFC: Prefrontal cortex
SPSS: Statistical package for the social sciences
STS: Superior temporal sulcus
TD: Typically developing
