# Supplementary material for: Spontaneous resting‐state gamma oscillations are not predictive of autistic traits in the general population
Source: Eur J Neurosci. 2018 Jun 19;48(8):2928–37. doi: 10.1111/ejn.13973 (PMC6220821; doi:10.1111/ejn.13973)
Supplement: Supplementary file 1 [file EJN-48-2928-s001.pdf]

EJN European Journal of Neuroscience FENS Federation of European Neuroscience Societies

Spontaneous Resting-State Gamma Oscillations are Not Predictive of Autistic Traits in the General Population

Kristel De Groot and Jan W. Van Strien

Review timeline:	Submission date:	04 November 2017
	Editorial Decision:	04 February 2018
	Revision Received:	25 Febuary 2018
	Editorial Decision:	18 April 2018
	Revision Received:	08 May 2018
	Accepted:	14 May 2018

Editor: John Foxe
Reviewer 1: Matthew Belmonte (The Com DEALL Trust, India)
Reviewer 2: Giorgio Di Lorenzo (University of Rome, Italy)

1st Editorial Decision 04 February 2018

Dear Ms. de Groot,

Your manuscript was reviewed by two external reviewers and the editors. The reviews indicate that there are several substantial issues that need to be clarified/resolved before we can consider your manuscript further for publication in EJN. First, the paper could benefit from substantial streamlining, which would give it more focus. There is a suggestion for a more informative title to the paper that should be considered and for additional relevant literature that should be considered. You will need to discuss the power of the study given the reporting of negative results. Can you also provide a better exposition of how spectral power was calculated. Other issues are listed in the appended reports below.

Please also attend to the following issues identified by our editorial office:
1. Ethical approval needs to be stated explicitly in the text.
2. Top of page 21: there is an erroneous paragraph break in the middle of a sentence.
3. All authors of cited papers should be included in the reference list.
4. Better resolution figures will be needed.

Thank you for submitting your work to EJN.

Kind regards,

John Foxe & Paul Bolam
co-Editors in Chief, EJN

Reviews:

Reviewer: 1

Comments to the Author
EJN-2017--11-25066: de Groot & al., "The Relationship Between Spontaneous Gamma Oscillations and Autistic Traits in the General Population"
<p>
The authors hypothesised associations of dimensional autistic traits, as measured by the Autism Spectrum Quotient (AQ), with increased gamma-band power, reduced left-dominant gamma power asymmetry, and decreased alpha power. In a sample of normal young adults, after accounting for effects of age and gender, they found no effect of gamma power, and association of AQ with GREATER left-dominant gamma asymmetry and GREATER alpha power, opposite to their hypotheses. These results are difficult to interpret and, as the authors note, tempered by the small amount of variance that they explain and by their sample's confounding of age with AQ. Nevertheless, these results contrary to prediction are interesting data that deserve to be reported, perhaps as a Short Communication.
<p>
The entire manuscript does read a bit verbose. The results, especially, are phrased repetitively and although this format does make for explicit clarity, some condensation might be achieved without loss of clarity. The introduction and discussion, too, range widely, and neither of the figures seems strictly necessary. There thus seems ample scope to abridge the manuscript without weakening its essential content.

EJN European Journal of Neuroscience FENS Federation of European Neuroscience Societies

<p>
The authors make much of their seeming finding that AQ scores increase with age. However, inferring any such longitudinal effect from a cross-sectional sample is fraught: the same approach could prove, for instance, that residents of east London are born with African or South Asian ethnicity and become caucasian as they age! The correlation between AQ and age is parsimoniously explained as a consequence of the authors' sampling strategy: they recruited university students, a population in whom the demographic abnormality of being a mature student can be a consequence of differences in personality and cognition. Thus the age/AQ relationship in this sample seems unlikely to generalise, especially when considered against the background of other studies' negative findings on this score.
<p>
The title seems uninformative: it says what was assayed but not what was found. For the findings one has to read into the abstract. Titles are of course a matter of individual style but if the authors want, they could use some more descriptive title such as "Left-Dominance but not Total Power of Resting Gamma EEG Predicts Autistic Traits in the General Population" - which, in just four words more than their current title, immediately conveys the essential findings.
<p>
The abstract's second sentence misleadingly asserts that "there have been no electrophysiological studies" of dimensional autistic traits. Rojas & al. (2011, cited elsewhere in the current manuscript) found decreased gamma phase-locking in parents of people with autism (who, as a group, are high in autistic traits), and there have indeed been electrophysiological studies of dimensional autistic traits using ERP measures since at least May 2013: see https://doi.org/10.1016/j.neuropsychologia.2013.03.002 and more recently http://dx.doi.org/10.1016/j.bpsc.2017.07.002 .
<p>
The abstract concludes that "the relationship between brain electrophysiology and pathological behaviour might not be strong enough to be detected in the general population". There are two issues with this conclusion: First, autistic traits outside the diagnosis are not definitively "pathological". If anything- and rather by definition! -such traits are normal individual variations. Second, other studies- in particular, each one of the two cited above- have indeed related brain electrophysiology to dimensional autistic traits. Likewise, autistic traits present less a "continuum of impairment" (page 3, line 13) than simply a "continuum".
<p>
Should the authors so desire, the data that they already have collected seem to present an opportunity to assay whether the result by Dickinson & al. (https://doi.org/10.1111/ejn.13645) on lower eyes-open resting alpha peak frequency in autistic versus non-autistic children extends dimensionally, and to this young-adult population.
<p>
The legend for Figure 2, "Band Mappings per Frequency Band for the Cz Electrode" doesn't make sense in this context of scalp maps: Scalp maps, inherently, map a field around a distributed electrode montage, not a single electrode. A single measurement at Cz cannot provide spatially extensive map information.
<p>
In sum, this is an interesting result, of possible interest to a general neuroscience audience but not straightforward in its interpretability, that might be delivered more pointedly and effectively in the form of a shorter communication.
<p>
Minor corrections:

Page 10, line 6: "participated in reward for course credit" should be "received course credit in reward for participation" or "participated in exchange for course credit"

Page 12, line 6: Delete the comma (,) immediately following "presented".

Page 12, line 15: Consider deleting "driven right leg", lest readers unfamiliar with the Biosemi ActiveTwo system receive the impression that the active electrode actually was placed on the right leg. In fact, because this paragraph's first sentence has established that an ActiveTwo was used, the CMS-DRL system is implicit and thus this entire sentence could be deleted.

Page 13, line 22: "advice" should be "advise"

Page 23, line 12: "groups itself" should be either "group itself" or "groups themselves"

Reviewer: 2

Comments to the Author
In the manuscript “EJN-2017-11-25066”, De Groot and Van Strien report the results of a quantitative EEG (qEEG) analysis of resting-state conditions (RSC) in a general population of young adults. They focused their attention on the relation between autistic traits (measured in the participants with a well-validated and largely used self-report scale) and EEG gamma band (previously reported increased and with a different asymmetry in RSC among subjects affected by autism). Accordingly to the Authors’ findings, in the sample no relation was observed between autistic trait dimensions and gamma power, as well as other EEG spectral bands.

Major comments

Despite the topic focused on this manuscript is worthy of research, I have some concerns about the methods implemented and, consequently, the results obtained. Below I list four methodological points that merit to be accurately taken into consideration.

Firstly, sample size calculation. Authors report in this manuscript a negative result and a possible reason may be the number of subjects recruited in the study. Because, in the manuscript, I have not found a description on a sample size calculation and comment in the “Discussion” section about the possible influence of sample size on the negative results, I have the suspect that Authors completely ignored this critical point. At least, Authors should discuss this point as a limit of their study and consider their findings as exploratory and discussed with caution. In fact, despite in the present study less than a hundred subjects were enrolled, I think that a research in the general population about relations between biological (in this case, neurophysiological) indices and personality / behavioral traits without a sample size has to be considered as exploratory.

Secondly, notch filter. Authors stated (page 12 of the manuscript / page 13 of the .pdf file) that "a notch filter of 50 Hz" was applied. Notch filter causes a distortion of oscillatory activity characteristics, particularly in gamma band. Authors did not consider this technical issue, maybe excluding a possible influence on their results. If they did not check for this effect, I strongly suggest to the Authors to reanalyze their data, applying more accurate methods to avoid this oscillatory distortion.

Thirdly, artifact correction. The method used to correct the eye movement artifacts does not follow the current standards in the field (as, for example, the independent component analysis, ICA). Despite widely used, the subtraction or regression methods are not able to separate accurately the non-cerebral signals from those signals originated from the brain (for examples see http://www.ncbi.nlm.nih.gov/pubmed/23087632). I suggest to the Authors to reanalyze the data implementing the ICA approach also to remove eye artifacts (and, consequently, for all artifact rejection). Otherwise, the Authors should discuss, in details, this limit on the current results, in the "Discussion" section.

Fourthly, EEG spectral power analysis. It is really not clear how Authors obtained the indices of EEG spectral power used in the statistical analysis. They have clearly to describe if an EEG spectral power average of the four (frontal, central, parietal, occipital) channel clusters (reported at page 14 of the manuscript / page 15 of the .pdf file) was calculated. Despite this, they have to indicate the reason for this choice respect to the information they might have derived separately by the four channel clusters. If on one hand the use of single index could give the opportunity to have a simplification of a phenomenon (in this case the gamma band oscillations), on the other hand with a reduction approach the variability of this oscillatory activities may be reduced, losing consequently the opportunity to observe association between gamma power in specific scalp topographic areas and autistic personality dimensions.

Moreover, Authors did not indicate if they used the absolute power or the relative power for the statistical analysis. It is mandatory to indicate this characteristic of EEG data analysis in the "Methods" section. Consequently, I strongly suggest to the Authors to reanalyse their data using (and reporting) both absolute and relative power indices, for all frequency band ranges in both conditions. A table containing descriptive statistics information on absolute and relative power could be inserted in the manuscript, also as "supplementary materials".

Finally, Authors should clarify the reason to use 0 Hz as the lowest value of frequency range for EEG delta activity. This is a really unusual value in the qEEG literature for subjects in a normal awake state.

Minor comments

- I suggest to the Authors to indicate in the "Title" the RSC as well as that the subjects were recruited "in the general population of young adults".

- In the "Abstract", no information about the sample is reported.

- I invite the Authors to be more cautious in considering that their qEEG results (with the several limits above described) can bring to suggest that (as reported by the Authors at the end of the "Abstract") "the relationship between brain electrophysiology and pathological behaviour might not be strong enough to be detected in the general population". In fact, other electrophysiological methods have to be used before to conclude about the real utility of neuro(psycho)physiology in exploring the relations between human brain physiology and the autistic traits.

- Authors prepared a comprehensive and well-written "Introduction" section, addressing the reader to their hypothesis. However, I believe it is too long.

- In the "Materials and methods" section, I invite the Authors to remove the title "Stimuli and Task" because in their data analysis there are only RSC. They could move the description of RSC in the "Procedure" paragraph. Moreover, Authors should indicate the period of the day (morning and / or afternoon, including the hour range) when the EEGs were recorded.

- Authors stated "EEG was recorded using a 32-channel amplifier" but they should clearly indicate if 32 electrodes where placed in the cap and used to acquire EEG activity. Looking at the Figure 2, it seems that the channels were 32.

- Authors report in "Results" section that gamma band power activities (both in low and high range, both in eyes open and eyes closed condition) had a non-normal distribution. Because this aspect has methodological consequences on statistical analysis, Authors carried out a robust approach with a bootstrapping procedure both in regression and correlation analyses. Was this the same for t-test used to compare men and women?

Moreover, Authors reported as effect size measure the partial eta squared: why did they use this measure respect to Cohen'd?

Finally, along with mean values (in the manuscript indicated as "M") Authors should report for gamma power also Standard Deviation values (in the manuscript indicated as "SD") for men and women.

- Because bootstrapping procedures are not common, please, in the statistical analysis Authors should explain the bias corrected accelerated (BCa), including also at least a reference for the method implemented. Moreover, they should indicate the value of confidence interval (90%, 95% or 99%) computed with the bootstrap method.

- Please, Authors should add information on the statistical analysis software implemented in the current research report.

- Looking at the correlation analysis, as expected based on qEEG literature of awake states, significant relations were present between EEG spectrum frequency bands. Consequently, as a general suggestion for an exploratory regression analysis (that could be presented as "supplementary materials"), in order to check the possible influence of other frequency bands on the regression findings presented in the manuscript, I propose to the Authors to insert in their hierarchical regression models after "age", "gender" and "gamma" variable also the other frequency bands, as separate variables.

- In the Tables, along with "Unstandardized Coefficients" "B" (in the manuscript table indicated as "b") Authors should report also "Standardized Coefficients" "Beta" (conventionally indicated as "ß"). Moreover, as for Table 3, Authors should include in the captions of Table 1 and Table 2 (or at the end of these tables as footnotes) the explanation of the acronyms "BCa CI".

- Autism is characterized by an alteration of brain connectivity and evidences from EEG/MEG researches (https://www.ncbi.nlm.nih.gov/pubmed/28467487) demonstrate clearly in autism functional dysconnectivity (both hypo- and hyper-connectivity) is present in several oscillation bands, including gamma oscillations. I encourage the Authors to discuss this topic in the "Discussion" Section because one possible future development in the study of autistic traits in the general population is the implementation of EEG/MEG functional connectivity techniques.

- I strongly suggest to the Authors to adding in "Discussion" Section a paragraph on study limitation as well as, at the end of the manuscript text (before the "Acknowledgements"), a "Conclusion" Section.

Authors' Response 25 February 2018

Subject: Submission of revised paper EJN-2017-11-25066

Dear editors,

Thank you for your email dated February 4 2018 enclosing the reviewers' comments. We thank you and the reviewers for the time and expertise invested in the comments. We have carefully read the reviews, and have revised the manuscript accordingly. Our responses are given in a point-by-point manner below. Also, as requested, any (major) changes in the manuscript were underlined so that these changes are easily identifiable for you and the reviewers.

We hope the revised version is now suitable for publication and look forward to hearing from you in due course. If the revised version is not yet suitable for publication, we would be happy to make any further changes required.

Sincerely,

Kristel de Groot and Jan van Strien

>>>

General comments by the editorial office

The paper could benefit from substantial streamlining, which would give it more focus.

> See our responses to both reviewers' comments on the length of the different sections.

You will need to discuss the power of the study given the reporting of negative results.

> Performing a power analysis is of course an essential aspect of any (empirical) research, regardless of whether the results are positive or negative. In fact, we did perform a power analysis. The outcome of this analysis is now reported in the participant section. For a more elaborate answer, see the response to the power comment by reviewer 2.

Can you also provide a better exposition of how spectral power was calculated.

> See our response to the spectral power question by reviewer 2. We now make explicit that absolute power was calculated.

There is a suggestion for a more informative title to the paper that should be considered.

> We thank both reviewers for their useful remarks on the title. Both remarks have been incorporated into a new title: "Spontaneous Resting-State Gamma Oscillations are Not Predictive of Autistic Traits in the General Population", which we agree is an improvement

over the old title.

There is a suggestion for additional relevant literature that should be considered.

> We thank both reviewers for suggesting additional literature. The suggestions were incorporated in the manuscript. We go into this issue in more depth in our responses to both reviewers.

Ethical approval needs to be stated explicitly in the text.

> Under “Participants” it is stated that “All procedures performed were in accordance with the ethics standards of the institutional review board and with the 1964 Helsinki declaration and its later amendments.”

Top of page 21: there is an erroneous paragraph break in the middle of a sentence.

> Solved. We apologise for this sloppy mistake.

All authors of cited papers should be included in the reference list.

> Solved. Hussman (2011) should be Hussman (2001). We apologise for this sloppy mistake.

Better resolution figures will be needed.

> Not relevant anymore since the Figures were removed in accordance with a comment from reviewer 1.

Please ensure that you provide a text and a figure file for the Graphical Abstract (as detailed in the instructions below).

> Done.

>>>

Reviewer 1

The entire manuscript does read a bit verbose. The results, especially, are phrased repetitively and although this format does make for explicit clarity, some condensation might be achieved without loss of clarity. The introduction and discussion, too, range widely, and neither of the figures seems strictly necessary. There thus seems ample scope to abridge the manuscript without weakening its essential content.

> We agree that the results are phrased repetitively, and that this indeed provides clarity, but also reads verbose. We changed the descriptive statistics to make it more concise. We did so by repeating similar findings in words, followed by the statistics from the four examined domains (eyes open/closed low/high gamma power). Applying this model to the remainder of the results section, however, proved to be difficult because of two reasons. First, combining findings makes it necessary to repeat statistics, which makes the format very unclear when dealing with longer statistics (such as the findings of multiple regression models). Second, because of minor differences between the sections, not everything could be easily repeated, making the format even less clear. Hence, we did not change the remainder of the results section. With regard to the Figures, we agree that the Figures are not necessary to convey the results properly (can be left out ‘without weakening its essential content’). We therefore excluded them. With regard to the introduction and discussion, we tried making it more concise as well, although in the end we mainly changed phrases that were specifically requested by one or both reviewers. This decision was partly fuelled by the comments of a previous reviewer, who found the introduction ‘exceptionally well written and thoroughly conceptualized’. We believe that, although a results section should always be direct and concise, sections such as the introduction and discussion can actually benefit from being more verbose, and that this is a matter of personal preference (see e.g. the opinion of the previous reviewer). In addition, a more elaborate introduction section makes the article more accessible to readers from autism research, who may need more background information on the biological mechanisms than the general neuroscience reader needs. We therefore only made minor adjustments to the conciseness of these sections.

The authors make much of their seeming finding that AQ scores increase with age. However, inferring any such longitudinal effect from a cross-sectional sample is fraught: the same approach could prove, for instance, that residents of east London are born with African or South Asian ethnicity and become caucasian as they age! The correlation between AQ and age is parsimoniously explained as a consequence of the authors' sampling strategy: they recruited university students, a population in whom the demographic abnormality of being a mature student can be a consequence of differences in personality and cognition. Thus the age/AQ relationship in this sample seems unlikely to generalise, especially when considered against the background of other studies' negative findings on this score.

> We thank the reviewer for this appropriate observation. It was incorporated in the manuscript, together with another

methodological/statistical issue that we noted when re-examining the data. Discussing both issues resulted in the following paragraph: “The present findings suggest the existence of a relationship between age and autistic traits in young TD adults. This outcome could be a by-product of methodological and statistical characteristics of the data. With regard to the former, the relationship between age and AQ could be explained by the sample itself. The higher level of autistic traits in older students could be a consequence of general differences in cognition and/or personality in this group. This is a common pitfall encountered when using cross-sectional data. With regard to the latter, thorough examination of the data shows that the relationship between age and AQ is characterised by heteroscedasticity, which is not necessarily bad, but which could invalidate the fitted models since they assume equal outcome variance across different values of the predictor. Together with the limited previous research on this matter, these problems make further investigation into the relationship between age and AQ essential.”

The title seems uninformative: it says what was assayed but not what was found. For the findings one has to read into the abstract. Titles are of course a matter of individual style but if the authors want, they could use some more descriptive title such as "Left-Dominance but not Total Power of Resting Gamma EEG Predicts Autistic Traits in the General Population" - which, in just four words more than their current title, immediately conveys the essential findings.

> We fully agree with the reviewer that a title indicative of the findings is generally much more useful than a more generic title. We thank the reviewer for the excellent suggestion concerning an alternative title. We changed the title according to suggestions by both reviewer 1 and 2, resulting in the following new title: “Spontaneous Resting-State Gamma Oscillations are Not Predictive of Autistic Traits in the General Population”. The reason we did not incorporate the left-dominance was that this (positive) finding was not robust.

The abstract's second sentence misleadingly asserts that "there have been no electrophysiological studies" of dimensional autistic traits. Rojas & al. (2011, cited elsewhere in the current manuscript) found decreased gamma phase-locking in parents of people with autism (who, as a group, are high in autistic traits), and there have indeed been electrophysiological studies of dimensional autistic traits using ERP measures since at least May 2013: see https://doi.org/10.1016/j.neuropsychologia.2013.03.002 and more recently http://dx.doi.org/10.1016/j.bpsc.2017.07.002.

> We have been unclear on this point: whereas there have been ERP studies, (de)synchronisation studies, and phase-locked activity studies, there have been no spontaneous resting-state studies into the broad autism spectrum (although spontaneous resting-state activity is, in fact, the easiest biomarker of all). We agree the previous phrasing was wrong, and thank the reviewer for noting this. We now state this more clearly in the manuscript (in abstract, introduction, and discussion). Examples of changed phrasings:
- Abstract: “While this idea is supported by neuroimaging studies, there have been few electrophysiological studies. Specifically, there have been no spontaneous resting-state studies yet.”
- Introduction: “In contrast to the discussed neuroimaging studies, electrophysiological methods such as electroencephalography (EEG) have been employed only sparsely to examine the possibility of a broad autism spectrum. In particular, some EEG measures, such as spontaneous resting-state activity, have not been employed at all in this area of research.”
- Discussion: “Because no previous research has related spontaneous resting-state EEG data to autistic traits in TD individuals, …”

The abstract concludes that "the relationship between brain electrophysiology and pathological behaviour might not be strong enough to be detected in the general population". There are two issues with this conclusion: First, autistic traits outside the diagnosis are not definitively "pathological". If anything- and rather by definition! -such traits are normal individual variations. Second, other studies- in particular, each one of the two cited above- have indeed related brain electrophysiology to dimensional autistic traits. Likewise, autistic traits present less a "continuum of impairment" (page 3, line 13) than simply a "continuum".

> We agree with the first remark that the term “pathological” is badly chosen, especially considering the fact that the present manuscript builds on the idea that such traits are indeed normal individual variations. Therefore, we changed the wording to: “… suggesting that the relationship between spontaneous resting-state brain electrophysiology and autistic traits might not be strong enough to be detected in the general population”. With regard to the second issue, as clarified in response to the previous comment, we did not mean general electrophysiological activity, but spontaneous resting-state activity. This has now been clarified throughout the manuscript. We again agree with the third commentary regarding the continuum. We agree with the chain of thought regarding autistic traits as natural, individual variation. Therefore, we excluded the impairment-part.

Should the authors so desire, the data that they already have collected seem to present an opportunity to assay whether the result by Dickinson & al. (https://doi.org/10.1111/ejn.13645) on lower eyes-open resting alpha peak frequency in autistic versus non-autistic children extends dimensionally, and to this young-adult population.

> We thank the reviewer for being so kind as to point to this recent publication. It certainly is an interesting study. However, we believe it crosses too many borders (both from a continuum approach to a clinical focus, and from an adult population to children) to be used as comparison.

The legend for Figure 2, "Band Mappings per Frequency Band for the Cz Electrode" doesn't make sense in this context of scalp maps: Scalp maps, inherently, map a field around a distributed electrode montage, not a single electrode. A single measurement at Cz cannot provide spatially extensive map information.

> We agree. However, we removed the Figures from the manuscript (see our response above in which we agree that the Figures do not add much value), hence making this comment not relevant anymore.

In sum, this is an interesting result, of possible interest to a general neuroscience audience but not straightforward in its interpretability, that might be delivered more pointedly and effectively in the form of a shorter communication.

> We hope we increased the readability of the manuscript by incorporating the comments by the reviewers.

Minor corrections:
Page 10, line 6: "participated in reward for course credit" should be "received course credit in reward for participation" or "participated in exchange for course credit".
Page 12, line 6: Delete the comma (,) immediately following "presented".
Page 12, line 15: Consider deleting "driven right leg", lest readers unfamiliar with the Biosemi ActiveTwo system receive the impression that the active electrode actually was placed on the right leg. In fact, because this paragraph's first sentence has established that an ActiveTwo was used, the CMS-DRL system is implicit and thus this entire sentence could be deleted.
Page 13, line 22: "advice" should be "advise".
Page 23, line 12: "groups itself" should be either "group itself" or "groups themselves".

> We thank the reviewer for noting these errors and for making these suggestions. We incorporated them all in the most recent version of the manuscript.

>>>

Reviewer 2

Firstly, sample size calculation. Authors report in this manuscript a negative result and a possible reason may be the number of subjects recruited in the study. Because, in the manuscript, I have not found a description on a sample size calculation and comment in the "Discussion" section about the possible influence of sample size on the negative results, I have the suspect that Authors completely ignored this critical point. At least, Authors should discuss this point as a limit of their study and consider their findings as exploratory and discussed with caution. In fact, despite in the present study less than a hundred subjects were enrolled, I think that a research in the general population about relations between biological (in this case, neurophysiological) indices and personality / behavioral traits without a sample size has to be considered as exploratory.

> We agree. Performing a power analysis is of course an essential aspect of any (empirical) research, regardless of whether the results are positive or negative. In fact, we did perform a power analysis. The outcome of this analysis is now reported in the participant section, and shows how the study was well-powered. The following can be found in the newest version of the manuscript: "A power analysis determined that this sample size was appropriate to achieve a medium-sized two-tailed power $(1 - \beta) > .85$ for the main analyses ($\alpha = .05$)". Since the sample size was sufficient, it does not form a limitation. Hence, we did not discuss it as such.

Secondly, notch filter. Authors stated (page 12 of the manuscript / page 13 of the .pdf file) that "a notch filter of 50 Hz" was applied. Notch filter causes a distortion of oscillatory activity characteristics, particularly in gamma band. Authors did not consider this technical issue, maybe excluding a possible influence on their results. If they did not check for this effect, I strongly suggest to the Authors to reanalyze their data, applying more accurate methods to avoid this oscillatory distortion.

> We thank the reviewer for this observation. We now include this consideration in the manuscript. We also re-analysed the data to see whether the notch filter indeed altered the findings. It did not, which we also discuss in the most recent version of the manuscript under "Robustness Checks": "With regard to the latter, analyses were re-run without applying a notch filter. Although the filter removes artefact caused by electrical power lines, it inherently causes a distortion of oscillatory activity, particularly in the gamma band. However, re-analysing the data without the notch filter did not change the conclusions." In addition, we now explain the need for such analyses in the "Analyses" section: "Fifth, a robustness check was performed. EEG (pre-)processing requires many analytical decisions. In the robustness check, it was examined whether the findings changed as a result of critical alternative decisions."

Thirdly, artifact correction. The method used to correct the eye movement artifacts does not follow the current standards in the field (as, for example, the independent component analysis, ICA). Despite widely used, the subtraction or regression methods are not able to separate accurately the non-cerebral signals from those signals originated from the brain (for examples see http://www.ncbi.nlm.nih.gov/pubmed/23087632). I suggest to the Authors to reanalyze the data implementing the ICA approach also to remove eye artifacts (and, consequently, for all artifact rejection). Otherwise, the Authors should discuss, in details, this limit on the current results, in the "Discussion" section.

> A broad range of artefact correction methods is used across the field. Although ICA is a more recent method, the traditional regression method used in the present manuscript is still widely used. There is no definitive support for either correction technique. Some articles (like the one mentioned by the reviewer) support ICA-like methods; others support regression-like methods (see e.g. https://www.ncbi.nlm.nih.gov/pubmed/15210288). In addition, the article referred to by the reviewer concerns combined EEG and

eye-tracking, which is different from the present situation because our participants did not perform saccades. Finally, it is our experience that ICA correction also influences other (higher) frequencies than those of eye movements, which is not desirable. This distortion is also shown by the study we refer to. We therefore respectfully disagree with the reviewer on this point.

Fourthly, EEG spectral power analysis. It is really not clear how Authors obtained the indices of EEG spectral power used in the statistical analysis. They have clearly to describe if an EEG spectral power average of the four (frontal, central, parietal, occipital) channel clusters (reported at page 14 of the manuscript / page 15 of the .pdf file) was calculated. Despite this, they have to indicate the reason for this choice respect to the information they might have derived separately by the four channel clusters. If on one hand the use of single index could give the opportunity to have a simplification of a phenomenon (in this case the gamma band oscillations), on the other hand with a reduction approach the variability of this oscillatory activities may be reduced, losing consequently the opportunity to observe association between gamma power in specific scalp topographic areas and autistic personality dimensions.

> If we understand this reviewer correctly, he/she would like (1) a more elaborate description of power calculation and (2) a rationale for the choice made regarding reporting the results for one single index versus the four clusters separately. More information on the power calculation was added to the “Electrophysiological Recordings and Signal Processing” section: “During the FFT algorithm, signals are transformed from the time domain to the frequency domain, dividing them into frequency components that all have their own distinctive amplitude and phase.” See also our next response on the power analysis.
Regarding the second issue, we agree that looking at the data per cluster might have provided some topographic information. The (now removed) Figure however, showed no major scalp differences for most frequency bands, indicating that analysing per cluster would not be very informative. Therefore, we opted for analysing the mentioned electrode clusters in total. Information on this was added to the manuscript: “As mentioned before, there were no significant topographical differences across the scalp for most frequency bands. Therefore, it was decided to analyse the clusters together.”
The validation for the chosen electrodes is mentioned in the manuscript: “Especially high frequency EEG (i.e. beta and gamma) is susceptible to myogenic artefacts. In previous research, the lowest beta and gamma spectral power has been observed at the midline, suggesting lower contamination of myogenic artefacts in those regions (Orekhova et al., 2007). Therefore, some studies advise focusing exclusively on the midline electrodes to minimise the contribution of myogenic artefacts. To include a broader range, we extended this to adjacent electrodes as well. Oscillatory activity was therefore pooled from the midline and adjacent electrodes: a frontal cluster (F3, Fz, F4), a central cluster (C3, Cz, C4), a parietal cluster (P3, Pz, P4), and an occipital cluster (O1, Oz, O2).”

Moreover, Authors did not indicate if they used the absolute power or the relative power for the statistical analysis. It is mandatory to indicate this characteristic of EEG data analysis in the “Methods” section. Consequently, I strongly suggest to the Authors to reanalyse their data using (and reporting) both absolute and relative power indices, for all frequency band ranges in both conditions. A table containing descriptive statistics information on absolute and relative power could be inserted in the manuscript, also as “supplementary materials”.

> We thank the reviewer for noting that we have not been specific enough with regard to how spectral power was calculated. We now explicitly mention (under Electrophysiological Recordings and Signal Processing) that absolute power was calculated. In addition, also as requested by the editor, we provided a better exposition of how spectral power was calculated: “During the FFT algorithm, signals are transformed from the time domain to the frequency domain, dividing them into frequency components that all have their own distinctive amplitude and phase.” We opted for using absolute power since relative power reduces individual variance in general power (and reduced variance has adverse impact on analyses). To accommodate for the reviewer’s concerns, we re-ran the analyses using relative power, and reported this in the “Robustness Checks” section: “Next to the filter robustness check, it was also examined whether the use of relative versus absolute power had any impact on the results. Therefore, all analyses were re-run using relative instead of absolute power. Again, none of the main findings and hence none of the conclusions changed.” In addition, we explain the need for such analyses in the “Analyses” section: “Fifth, a robustness check was performed. EEG (pre-)processing requires many analytical decisions. In the robustness check, it was examined whether the findings changed as a result of critical alternative decisions.”

Finally, Authors should clarify the reason to use 0 Hz as the lowest value of frequency range for EEG delta activity. This is a really unusual value in the qEEG literature for subjects in a normal awake state.

> We thank the reviewer for noting this. In fact, the lowest value has never been 0 Hz, since a low cut-off of 0.1 Hz was used. We do appreciate the comment that this as well may be an unusual value for awake subjects. We now include this consideration in the manuscript. We also re-analysed the data to see whether the findings changed when using a higher low cut-off of 0.5 Hz. It did not, which we also discuss in the most recent version of the manuscript under “Robustness Checks”: “With regard to the former, low frequency activity (such as 0.1 Hz) is theoretically possible, but unusual in individuals in a normal awake state. Therefore, the analyses were re-run using a higher low cut-off: 0.5 Hz. None of conclusions changed.” In addition, we explain the need for such analyses in the “Analyses” section: “Fifth, a robustness check was performed. EEG (pre-)processing requires many analytical decisions. In the robustness check, it was examined whether the findings changed as a result of critical alternative decisions.”

Minor comments:

I suggest to the Authors to indicate in the “Title” the RSC as well as that the subjects were recruited “in the general population of young adults”.

> We included the nature of the measurement (resting-state) in the title, as well as the population (TD). We changed the title according to suggestions by both reviewer 1 and 2, resulting in the following new title: “Spontaneous Resting-State Gamma Oscillations are Not Predictive of Autistic Traits in the General Population”. We did not add “of young adults”, since this would make the title relatively long (17 words).

In the “Abstract”, no information about the sample is reported.

> We thank the reviewer for noting this. We added information on the sample size and the age group, resulting in the following paraphrase: “In order to examine the autism spectrum hypothesis, the present study tried to predict the level of autistic traits typically developing young adults (n = 93) exhibit …”.

I invite the Authors to be more cautious in considering that their qEEG results (with the several limits above described) can bring to suggest that (as reported by the Authors at the end of the “Abstract”) “the relationship between brain electrophysiology and pathological behaviour might not be strong enough to be detected in the general population”. In fact, other electrophysiological methods have to be used before to conclude about the real utility of neuro(psycho)physiology in exploring the relations between human brain physiology and the autistic traits.

> We agree that we should be more cautious with this phrasing considering previous findings using alternative electrophysiological methods (such as phase-locked or event-related EEG). We received a similar argument from the other reviewer. We now specify what kind of brain electrophysiology we are talking about (since we are the first to examine that in a TD population): “…, suggesting that the relationship between spontaneous resting-state brain electrophysiology and autistic traits might not be strong enough to be detected in the general population.” This is now correct phrasing since it is better specified. In addition, in line with other comments, we now discuss other electrophysiological measures (such as connectivity measures) as possible future research avenues.

Authors prepared a comprehensive and well-written “Introduction” section, addressing the reader to their hypothesis. However, I believe it is too long.

> We want to thank the reviewer for the compliment. We tried making the introduction more concise, but in the end, we mainly changed phrases that were specifically requested by one or both reviewers. This decision was also fuelled by the comments of a previous reviewer, who found the introduction ‘exceptionally well written and thoroughly conceptualized’. Introduction length and level of elaborateness is always a matter of personal preference. We believe that the present introduction benefits from being more elaborate to include a broader readership (e.g. individuals who are not that familiar with electrophysiology but are specialised in autism). In addition, since the introduction reads well, we believe there is little cost. We did make the results section more concise.

In the “Materials and methods” section, I invite the Authors to remove the title “Stimuli and Task” because in their data analysis there are only RSC. They could move the description of RSC in the “Procedure” paragraph. Moreover, Authors should indicate the period of the day (morning and / or afternoon, including the hour range) when the EEGs were recorded.

> We thank the reviewer for this suggestion. As suggested, we moved the information under “Stimuli and Task” to “Procedure”, hereby creating a more logical flow. In addition, we specified the starting times of the experimental sessions: “Sessions either started at 9:00 am, 11:00 am, 1:00 pm, or 3:00 pm.”

Authors stated “EEG was recorded using a 32-channel amplifier” but they should clearly indicate if 32 electrodes where placed in the cap and used to acquire EEG activity. Looking at the Figure 2, it seems that the channels were 32.

> This is clarified in the most recent version of the manuscript: “A number of 32 Ag/AgCl active electrodes were placed on the scalp by means of a head cap according to the 10-20 placing system.”

Authors report in “Results” section that gamma band power activities (both in low and high range, both in eyes open and eyes closed condition) had a non-normal distribution. Because this aspect has methodological consequences on statistical analysis, Authors carried out a robust approach with a bootstrapping procedure both in regression and correlation analyses. Was this the same for t-test used to compare men and women?

> We thank the reviewer for noting this. We re-ran the analyses on gender and age involving spectral data using bootstrapping. These results are now reported in the descriptive statistics: “None of the mean values significantly differed between men and women, $t(91) = 0.19$, $p = .844$, $\eta_{2p} < .01$ (eyes open low gamma), $t(91) = -0.03$, $p = .974$, $\eta_{2p} < .01$ (eyes closed low gamma), $t(91) = 0.63$, $p = .508$, $\eta_{2p} < .01$ (eyes open high gamma), and $t(91) = 0.37$, $p = .699$, $\eta_{2p} < .01$ (eyes closed high gamma). Finally, power did not significantly correlate with age, $r = .07$ (bootstrap bias .011), $p = .521$ (eyes open low gamma), $r = .11$ (bootstrap bias .017), $p = .318$ (eyes closed low gamma), $r = .10$ (bootstrap bias .008), $p = .353$ (eyes open high gamma), $r = .11$ (bootstrap bias .009), $p = .284$ (eyes closed high gamma).”

Moreover, Authors reported as effect size measure the partial eta squared: why did they use this measure respect to Cohen’d?

> The present choice for the partial eta squared was not driven by theoretical arguments, but purely by practicality: the used statistical program (SPSS) does not output d, but does output eta. These effect sizes are conceptually equal. By using the effect size that is outputted by the used programme, we prevented additional out-of-programme (or different-programme) math, which decreases the chance at mistakes.

Finally, along with mean values (in the manuscript indicated as “M”) Authors should report for gamma power also Standard Deviation values (in the manuscript indicated as “SD”) for men and women.

> In the newest version of the manuscript, we leave out the separate means entirely, since both reviewers asked for a more concise results section (which we did for the descriptive statistics). This was decided since the scores from men and women did not differ from each other (not significantly and in many cases not even when rounding at 2 decimal places).

Because bootstrapping procedures are not common, please, in the statistical analysis Authors should explain the bias corrected accelerated (BCa), including also at least a reference for the method implemented. Moreover, they should indicate the value of confidence interval (90%, 95% or 99%) computed with the bootstrap method.

> We added information on BCa, including a reference: “Analyses involving spectral data were made robust against their inherent skewness by using bootstrapping (1000 samples), a technique that estimates the sampling distribution of a statistic by taking repeated samples from a dataset (Efron, 1987).” In addition, the 95% used is now explicitly mentioned in Table 1 and 2, and in the note of Table 3.

Please, Authors should add information on the statistical analysis software implemented in the current research report.

> Resolved. The analyses section now starts with: “After pre-processing the data, the final analyses were performed using IBM SPSS version 24.”

Looking at the correlation analysis, as expected based on qEEG literature of awake states, significant relations were present between EEG spectrum frequency bands. Consequently, as a general suggestion for an exploratory regression analysis (that could be presented as “supplementary materials”), in order to check the possible influence of other frequency bands on the regression findings presented in the manuscript, I propose to the Authors to insert in their hierarchical regression models after “age”, “gender” and “gamma” variable also the other frequency bands, as separate variables.

> While we understand the reviewer’s viewpoint, we think that a number of more than 3 predictors would be too much for the present sample size. Adding delta, theta, alpha, and beta would mean a total number of regression predictors of 7. A post-hoc power analysis indicated that the power would in that case drop to .75, which is quite low.

In the Tables, along with “Unstandardized Coefficients” “B” (in the manuscript table indicated as “b”) Authors should report also “Standardized Coefficients” “Beta” (conventionally indicated as “ß”).

> We believe in this case this does not provide much additional information, since the used variables are easy to interpret. Also, it complicates the (now easy and quick to understand) Tables.

Moreover, as for Table 3, Authors should include in the captions of Table 1 and Table 2 (or at the end of these tables as footnotes) the explanation of the acronyms “BCa CI”.

> This is indeed very informative. A footnote was added to both Tables: “Note: N = 93; BCa CI = bias corrected accelerated confidence interval.”

Autism is characterized by an alteration of brain connectivity and evidences from EEG/MEG researches (https://www.ncbi.nlm.nih.gov/pubmed/28467487) demonstrate clearly in autism functional dysconnectivity (both hypo- and hyper-connectivity) is present in several oscillation bands, including gamma oscillations. I encourage the Authors to discuss this topic in the “Discussion” Section because one possible future development in the study of autistic traits in the general population is the implementation of EEG/MEG functional connectivity techniques.

> We thank the reviewer for this literature suggestion. This is indeed an interesting topic to discuss. We implemented it in the following way: “A variation on this concerns examining brain connectivity, which differs across frequencies, with potential over-connectivity in the higher frequency bands of individuals diagnosed with ASD. Indeed, connectivity has been shown to be altered in clinical autism populations as measured with EEG/MEG (for an overview, see O’Reilly et al., 2017). So far, this is supported in a TD population using fMRI (Di Martino et al., 2009), leaving the relationship between electrophysiologically measured connectivity and autistic traits across the broad autism spectrum unanswered.”

I strongly suggest to the Authors to adding in “Discussion” Section a paragraph on study limitation as well as, at the end of the

manuscript text (before the “Acknowledgements”), a “Conclusion” Section.

> We did add limitations to the discussion, but did not add special sections to the discussion because we think it would impair the current flow of the discussion.

2nd Editorial Decision 18 April 2018

Dear Dr. de Groot,

Your revised manuscript was re-evaluated by one of the external reviewers as well as by the Editorial team and we are pleased to inform you that it will be accepted for publication in EJN after a few very minor revisions. The reviewer raises a few minor points that need to be addressed and there are few issues that we have identified (see below). Once done, we will send it off to the Production Editors.

- An explicit statement of ethical approval still needed.
- All authors of cited papers should be included in the reference list
- First reference in the list has an extraneous "author" at the end.

Thank you for submitting your work to EJN.

Best wishes,

Paul Bolam & John Foxe
co-Editors in Chief, EJN

Reviews:

Reviewer: 1

Comments to the Author
The authors have satisfactorily addressed or rebutted all points raised by the reviewers. I offer two suggestions and one minor correction all of which can be addressed without further review:

In the abstract, append to "higher age being related to more autistic traits" the important and perhaps crucial qualification "within this student population".

In the final sentence of the abstract, change the definite "the relationship" to the indefinite "any relationship", to reflect the negative nature of the current result.

Page 11, line 12: "alfa" should be "alpha"

Authors’ Response 08 May 2018

Subject: Submission of revised paper EJN-2017-11-25066.R1

Dear editors,

Thank you for your email dated April 18 2018 in response to our revised version of the manuscript. We thank you and the reviewer for the final comments. We have revised the manuscript in accordance with the comments. Our responses are given in a point-by-point manner below.

We hope the revised version is now suitable for publication and look forward to hearing from you in due course.

Sincerely,

Kristel de Groot and Jan van Strien

>>>

1. An explicit statement of ethical approval still needed.

An explicit statement of ethical approval was added at the end of the manuscript: “Statement of Ethical Approval. Informed consent was obtained from all participants included in the study. The research proposal was reviewed by the Ethical Committee of the Department of Psychology, Education & Child Studies of the Erasmus University Rotterdam. All procedures performed were in accordance with the 1964 Helsinki Declaration and its later amendments.”

2. All authors of cited papers should be included in the reference list.

All authors are now included in the reference list.

3. First reference in the list has an extraneous "author" at the end.

We believe the present format is an appropriate way of referring to the DSM. Since the author of the work is also the publisher, repeating “American Psychiatric Association” is redundant. It is sufficient to refer back to the author. Therefore, instead of the name of a publisher, the reference ends with the word “author”. This is in line with APA policy. Some use “American Psychiatric Publishing”, the publishing house. However, since this publishing house is owned by the APA, both “author” and “American Psychiatric Publishing” are correct.

4. In the abstract, append to "higher age being related to more autistic traits" the important and perhaps crucial qualification "within this student population".

Changed into the reviewer’s suggestion.

5. In the final sentence of the abstract, change the definite "the relationship" to the indefinite "any relationship", to reflect the negative nature of the current result.

Changed into the reviewer’s suggestion.

6. Page 11, line 12: "alfa" should be "alpha".

Corrected. Thank you for noticing this.
